# Deficiencies in reporting results of lesbians and gays after donor intrauterine insemination and assisted reproductive technology treatments: a review of the first emerging studies

**DOI:** 10.1186/s12958-015-0053-9

**Published:** 2015-05-29

**Authors:** Juan J. Tarín, Miguel A. García-Pérez, Antonio Cano

**Affiliations:** Department of Functional Biology and Physical Anthropology, Faculty of Biological Sciences, University of Valencia, Burjassot, Valencia, 46100 Spain; Department of Genetics, Faculty of Biological Sciences, University of Valencia, Burjassot, Valencia, 46100 Spain; Research Unit-INCLIVA, Hospital Clínico de Valencia, Valencia, 46010 Spain; Department of Pediatrics, Obstetrics and Gynecology, Faculty of Medicine, University of Valencia, Valencia, 46010 Spain; Service of Obstetrics and Gynecology, University Clinic Hospital, Valencia, 46010 Spain

**Keywords:** Cause of infertility, Donor intrauterine insemination, In-vitro fertilization, Oocyte-donation cycles, Semen parameters, Sexual orientation, Gestational surrogacy

## Abstract

At a time when increasing numbers of lesbians and gays consider parenthood using reproductive assistance in infertility centers, the present review aims to summarize the results obtained so far by lesbians after intrauterine insemination (IUI) and in-vitro fertilization (IVF) using donor spermatozoa (D-IUI and D-IVF, respectively) and gays entering into gestational-surrogacy programs. Data show that gays display normal semen parameters and lesbians exhibit no specific causes of female infertility except perhaps for polycystic ovary syndrome (PCOS) and some PCOS-related factors. Pair-bonded lesbians entering into D-IUI programs, tend to have higher pregnancy and delivery percentages following spontaneous or induced ovulation than single or pair-bound heterosexual women. The only single study reporting success percentages of lesbians after D-IVF provides, however, puzzling results. In particular, pair-bonded lesbians have lower pregnancy and live-birth percentages than pair-bonded heterosexual women in fresh D-IVF cycles but percentages are similar in frozen/thawed D-IVF cycles. Like in lesbians after D-IUI, surrogate women recruited by pair-bonded gays/single men tend to have higher pregnancy percentages and lower miscarriage percentages than surrogate women recruited by heterosexual couples. Notably, all the reports reviewed in the present study are methodologically flawed because of sampling bias, small sample sizes and inadequate use of statistical methods to control for the effects of influential covariates including age, smoking habits, previous gynecological problems, hormonal stimulation type and protocol, and number of prior treatment types and pregnancies/deliveries. Clinicians, reproductive biologists and editors of fertility/infertility journals should make efforts to prevent these deficiencies in future data reporting.

## Background

It has been reported that gays and lesbians reproduce significantly less than heterosexual subjects. For instance in one U.S. random-sample survey performed in 1994, only 27 % of men who identified as homosexual said they were fathers, compared with 60 % of other men. Differences between groups were smaller in women but the percentage was also lower in lesbians (67 % versus 72 % in other women) (for review, see [1]). Iemmola and Camperio-Ciani [2] found in an Italian survey that non-heterosexual men (bisexuals were also included in this group) had significantly fewer children than heterosexual men (an average of 0.12 versus 0.58 children/man). Dissimilarities were even larger in a British study [3] with an average of 0.002 children/man in exclusive white homosexuals versus 0.36 children/man in white heterosexuals.

Despite differences in number of children fathered, there are no reasons to think that the potential for reproduction of homosexuals is decreased when compared with heterosexuals. Firstly, sexual preference is governed by the brain independently of plasma concentrations of sex hormones as shown in the mouse model [4]. Likewise, plasmatic/salivary levels of sex hormones are not associated with sexual orientation in human beings (see later). Secondly, there are more obvious reasons than decreased potential for reproduction to explain discrepancies in number of children fathered between homosexuals and heterosexuals. For instance, homosexuals do not engage in sexual activities with individuals of the opposite sex as frequently as heterosexuals.

Thanks to the social changes taking place in Western society with respect to gay rights increasing numbers of lesbians and gays seek reproductive assistance to achieve parenthood in infertility centers. Consequently, a number of studies reporting success percentages of lesbians after intrauterine insemination (IUI) and in-vitro fertilization (IVF) using donor spermatozoa (D-IUI and D-IVF, respectively) and gays entering into gestational-surrogacy programs are starting to emerge.

A priori, success percentages of lesbians and gays should be similar or even higher than those exhibited by heterosexuals attending infertility clinics. Actually, although some studies suggest that some lesbians have higher testosterone levels than heterosexual women, especially those that adopt a masculine role in their couple relationship [5, 6], most studies find no significant differences between homosexual and heterosexual subjects in adult plasmatic/salivary levels of sex hormones and gonadotropins, and secretion pattern of gonadotropins ([7–10] for reviews see [11–13]). In addition, pair-bonded lesbians usually enter into D-IUI and/or D-IVF programs to fulfill their wishes to get pregnant and have their own offspring, not because they are infertile. In contrast, most women from heterosexual couples attend reproductive health clinics to undergo D-IUI and/or D-IVF because their partner is an infertile man, human immune virus (HIV)-positive, or carries another sexually transmitted disease or a genetic disease. Of note, women with an infertile partner may be as well subfertile, especially when their partner suffers from oligozoospermia [14]. Moreover, lesbians may have a higher incidence of polycystic ovary syndrome (PCOS) and some PCOS-related factors including polycystic ovaries (see later), circumstances that may increase further the success percentages after D-IUI and/or D-IVF. Indeed, women with PCOS going through IUI [15–17] and women with polycystic ovaries undergoing IVF and/or intracytoplasmic sperm injection (ICSI) treatment [18–20] exhibit increased pregnancy and live-birth/delivery percentages.

Likewise pair-bonded gays usually enter into gestational surrogacy programs to fulfill their desire to raise children, create families and pass on their genes to offspring (for references, see [21]), not because they are infertile. In contrast, pair-bonded heterosexual men use these programs because their partner (a) has previously experienced repeated implantation failure, unexplained or failed treatment for recurrent pregnancy loss, or poor obstetrical history; (b) has no uterus (congenital or post-hysterectomy) or a severe Müllerian anomaly; (c) suffers from a medical condition where pregnancy could pose a significant health risk; or (d) takes medications that are or could potentially be teratogenic [22]. Note that some of these conditions in particular, recurrent pregnancy loss [23–25] and repeated implantation failure (for review, see [26]) not only may be associated with female factors but also with male factors.

At a time when increasing numbers of lesbians and gays consider parenthood using reproductive assistance in infertility centers [22] the present review aims to summarize the results obtained so far by lesbians after D-IUI and D-IVF and gays entering into gestational-surrogacy programs. This information may be helpful not only to future intended homosexual parents seeking reproductive assistance in infertility centers but also to clinicians and reproductive biologists assisting this collective. We are aware that human sexual behavior is highly complex and variable and sexuality is much more complex and individual than a simpler bipolar (heterosexual versus homosexual) classification [27]. However, for the sake of simplicity and pragmatism, we use the traditional bipolar classification.

## Methods

A literature review based on publications up to February 2015 identified by PubMed database searches using the following key words: lesbians, gays, homosexuals, heterosexuals, sexual orientation, fertility, infertility, gestational surrogacy, reproductive fitness, intrauterine insemination, in vitro fertilization, testosterone, estradiol, steroids, hormone profiles, gonadotropins, oligozoospermia, teratozoospermia, asthenozoospermia, normozoospermia. In addition, a hand search was done to explore the references cited in the primary articles. This literature search evidenced 4 and 2 articles reporting success percentages of lesbians and gays, respectively, after D-IUI and assisted reproductive technology treatments (see Tables 1 and 2). All these articles were analyzed without applying inclusion or exclusion criteria.

**Table 1:** Success percentages of lesbians after D-IUI, fresh D-IVF and frozen/thawed D-IVF

Treatment	Outcome	Ovarian stimulation	Lesbians	Pair-bonded lesbians	Pair-bonded heterosexual women	Single women	Single heterosexual women	*P* value	Reference
D-IUI	Pregnancy per cycle	None		22.2 (10/45)^a^		8.8 (19/217)		0.016^b^	Ferrara *et al.* [30]
		hMG		20.8 (5/24)		10.4 (14/134)		0.172^b^	Ferrara *et al.* [30]
		CC		7.2 (5/69)		5.4 (10/185)		0.560^b^	Ferrara *et al.* [30]
		None, CC or hMG	13.5 (26/192)				9.6 (83/864)	0.105^c^	Ferrara *et al.* [38]
		None, CC, FSH or CC+ FSH		20.5 (90/438)	14.8 (44/298)			0.046^c^	Nordqvist *et al.* [29]
	Ongoing pregnancy per woman after a mean number of 3 cycles	None, CC or gonadotropins		≈60 % (≈72/120)^d^	≈55 % (≈72/131)^d^			0.203^c^	De Sutter *et al.* [33]
	Pregnancy per woman after a mean number of ≈ 3 cycles^e^	None, CC, FSH or CC+ FSH		61.6 (90/146)	44.0 (44/100)			0.006^c^	Nordqvist *et al.* [29]
	Live-birth per cycle	None, CC, FSH or CC+ FSH		16.0 (70/438)	12.8 (38/298)			0.224^c^	Nordqvist *et al.* [29]
	Live-birth per woman after a mean number of ≈ 3 cycles^e^	None, CC, FSH or CC+ FSH		48.0 (70/146)	38.0 (38/100)			0.123^c^	Nordqvist *et al.* [29]
Fresh D-IVF	Pregnancy per cycle	Not specified		34.6 (44/127)	45.2 (57/126)			0.085^c^	Nordqvist *et al.* [29]
	Pregnancy per woman after a mean number of ≈ 1.5 cycles^f^	Not specified		47.8 (44/92)	68.7 (57/83)			0.005^c^	Nordqvist *et al.* [29]
	Live-birth per cycle	Not specified		27.6 (35/127)	33.3 (42/126)			0.318^c^	Nordqvist *et al.* [29]
	Live-birth per woman after a mean number of ≈ 1.5 cycles^f^	Not specified		38.0 (35/92)	50.6 (42/83)			0.095^c^	Nordqvist *et al.* [29]
Frozen/thawed D-IVF	Pregnancy per cycle	Not specified		31.6 (31/98)	31.7 (33/104)			0.988^c^	Nordqvist *et al.* [29]
	Pregnancy per woman after a mean number of ≈ 2 cycles^g^	Not specified		63.3 (31/49)	64.7 (33/51)			0.881^c^	Nordqvist *et al.* [29]
	Live-birth per cycle	Not specified		24.5 (24/98)	23.1 (24/104)			0.814^c^	Nordqvist *et al.* [29]
	Live-birth per woman after a mean number of ≈ 2 cycles^g^	Not specified		49.0 (24/49)	47.1 (24/51)			0.848^c^	Nordqvist *et al.* [29]

^a^Counts used to calculate percentages are shown in parentheses

^b^2 × 2 contingency Fisher’s exact test

^c^2 × 2 contingency Pearson’s chi-square test

^d^Data were represented in a graphical format. Thus, we do not know the exact number of women with an ongoing pregnancy

^e^3.00 (438/146) and 2.98 (298/100) D-IUI cycles per woman in the lesbian and heterosexual group, respectively

^f^1.38 (127/92) and 1.52 (126/83) D-IVF cycles per woman in the lesbian and heterosexual group, respectively

^g^2.00 (98/49) and 2.04 (104/51) D-IVF cycles per woman in the lesbian and heterosexual group, respectively

**Table 2:** Success percentages of gays entering into gestational-surrogacy programs

Treatment	Outcome	Pair-bonded gays/single men	Heterosexual couples	“Failed to carry” heterosexual couples^a^	“Cannot carry pregnancy” hererosexual couples^b^	P value	Reference
Gestational-surrogacy program	Delivery per surrogate woman after a maximum of either two fresh cycles or one fresh and one frozen transfer cycle	55.6 (25/45)^c^	48.9 (23/47)			0.525^d^	Grover *et al.* [21]
	Pregnancy per cycle^f^	59.7 (37/62)		50.0 (66/132)	54.0 (75/139)	0.447^e^	Dar *et al.* [22]
	Miscarriage per pregnant surrogate woman	10.8 (4/37)		25.8 (17/66)	20.0 % (15/75)	0.193^e^	Dar *et al.* [22]

^a^96 patients suffering from recurrent implantation failure (n = 57), recurrent pregnancy loss (n = 30) and previous poor pregnancy outcome (n = 9)

^b^108 patients suffering from uterine malformations/Asherman’s syndrome (n = 34), Müllerian agenesis (Mayer–Rokitansky–Kuster–Hauser syndrome; n = 33) and maternal medical conditions precluding pregnancy (n = 41)

^c^Counts used to calculate percentages are shown in parentheses

^d^2 × 2 contingency Pearson’s chi-square test

^e^3 × 2 contingency Pearson’s chi-square test

^f^A cycle was defined as one stimulation cycle with fresh transfer and any subsequent frozen embryo transfer from the same cycle

For the sake of uniformity, data from 2 × 2 and 3 × 2 contingency tables were re-analyzed using Pearson's chi-square test. Fisher’s exact test was applied in the analysis of 2 × 2 contingency tables if the expected values in any of the cells were less than 5. Data re-analysis was performed using the IBM SPSS Statistics, Version 22 (© Copyright IBM corporation *et al.* 1989, 2013). P values from tests comparing continuous variables were kept in their original form. Significance was defined as P ≤ 0.05.

### Causes of infertility in lesbians

Despite a higher prevalence of *Chlamydia* infection [28, 29], the incidence of tubal-factor infertility in lesbians is similar to that found in heterosexual women [29–31]. However, two articles [10, 31] have reported that, compared with heterosexual women, lesbians may exhibit higher prevalence of PCOS and some PCOS-related factors including polycystic ovaries, hirsutism, obesity, and higher testosterone and androstenedione levels. A third study [32] has shown that although lesbians with PCOS display higher body mass index than heterosexual women with PCOS, they have similar hyperandrogenism-related clinical or biochemical characteristics. Likewise, other studies [29, 33] have found no significant differences in incidence of PCOS and polycystic ovaries between lesbians and heterosexual women.

### Seminal parameters in gays

There is a total absence of current studies reporting seminal parameters in gays. Just a limited number of articles published in the 60’s and 70’s decades dealt with this topic. Most of them evidenced no significant differences in semen parameters between homosexual and heterosexual men [34–36]. Only one article by Kolodny *et al.* [37] reported the presence of significant differences in sperm counts among groups of homosexuals established according to the Kinsey’s heterosexual-homosexual rating scale. In particular, azoospermia and oligozoospermia were concentrated in men with ratings 5 or 6 on the Kinsey’s scale, i.e., men almost exclusively or exclusively homosexuals, respectively. These data, however, should be taken with caution because Kolodny *et al.* [37] did not control for several covariates that may influence seminal parameters such as health status, use of psychotropic drugs, and degree of physical exercise and sexual activity. In addition, the Kinsey’s rating procedure did not discriminate between overt behavior only or the subjects’ general sexual responsiveness including attraction and fantasies.

### Success percentages of lesbians after D-IUI and D-IVF

#### D-IUI

Table 1 shows that lesbians tend to have higher success percentages than heterosexual women after D-IUI. Ferrara *et al.* [30] reported a significantly higher pregnancy percentage per D-IUI cycle in pair-bonded lesbians following spontaneous ovulation than in single women (22.2 %, versus 8.8 %). After ovarian stimulation with gonadotropins (20.8 % versus 10.4 %) or clomiphene citrate (7.2 % versus 5.4 %), pair-bonded lesbians still showed higher pregnancy percentage per cycle although differences were not significant. Differences between groups may be explained by the younger age of pair-bonded lesbians (mean age: 34.5 years; range: 26–44 years) compared with single women (mean age: 38.5; range: 29–47 years; P ≤ 0.005). Furthermore, some single women may have had a previous infertile relationship with a heterosexual partner and, therefore, at least some of them may have been subfertile. We should note, however, that pair-bonded lesbians under 35 years of age exhibited higher, although not statistically significant, pregnancy percentage per D-IUI cycle than single women from the same age group (23.9 %, 16/67 versus 11.5 %, 7/61; Pearson’s chi-square test: P ≤ 0.068). Likewise, in women 30–35 years old, miscarriage percentage per pregnant woman was significantly lower in pair-bonded lesbians (10.0 %, 1/10 versus 66.7 %, 4/6 in single women; Fisher’s exact test: P ≤ 0.036). Noteworthy, differences between groups may have been even higher if we take into account that some lesbians may have been included incorrectly in the group of single women. A later study from the same group [38] reported also a higher, although not significant, pregnancy percentage per D-IUI cycle in lesbians compared with single heterosexual women (13.5 % versus 9.6 %). Of note, Ferrara *et al.* [38] on this occasion did not specify whether or not lesbians were pair-bonded and did not compare the effect of ovarian stimulation on pregnancy percentage between lesbians and heterosexual women.

Another study by De Sutter *et al.* [33] evidenced a non-significant higher ongoing pregnancy percentage per woman in pair-bonded lesbians than in pair-bonded heterosexual women (≈60 % versus ≈ 55 %) after a mean number of 3 D-IUI cycles. Notwithstanding, this study did not either control for women’s age (mean age: 30.6 years in lesbians versus 31.9 years in heterosexuals) and hormonal stimulation [none, clomiphene citrate (CC) or gonadotropins]. We should bear in mind that, in contrast to Pearson’s chi-square test, binary logistic regression models allow you to control for categorical, interval or continuous covariates.

More recently, Nordqvist *et al.* [29] have reported a significantly higher pregnancy percentage per D-IUI cycle (20.5 % versus 14.8 %) and per woman after a mean number of ≈ 3 D-IUI cycles (61.6 % versus 44.0 %) in pair-bonded lesbians compared with pair-bonded heterosexual women. Live-birth percentage per D-IUI cycle (16.0 % versus 12.8 %) and per woman after a mean number of ≈ 3 D-IUI cycles (48.0 % versus 38.0 %) were also higher in the homosexual group, although differences were not significant. Like in the other previous studies, Nordqvist *et al.* [29] did not control for influential covariates including: (1) hormonal stimulation [none, CC, follicle stimulating hormone (FSH) or CC+ FSH]; (2) smoking habits (2.8 % of lesbians and 9.2 % of heterosexuals were smokers); (3) previous gynecological problems (the incidence of several previous gynecological problems in lesbians was at least twice as high as in heterosexuals); (4) number of prior treatments (lesbians had previously undergone D-IUI and D-IVF more often than heterosexuals, whereas heterosexuals had previously undergone ICSI with partner sperm more often than lesbians); and (5) previous pregnancies/deliveries (heterosexuals exhibited higher incidence of previous deliveries than lesbians). Women’s age was, however, homogenous in both groups (mean ± standard deviation: 32.4 ± 4 years).

### D-IVF

Nordqvist *et al.* [29] compared success percentages between pair-bonded lesbians and pair-bonded heterosexual women after fresh and frozen/thawed D-IVF (Table 1). On this occasion, pregnancy percentage per fresh D-IVF cycle (34.6 % versus 45.2 %) and per woman after a mean number of ≈ 1.5 fresh D-IVF cycles (47.8 % versus 68.7 %) were lower in the lesbian group. Live-birth percentage per fresh D-IVF cycle (27.6 %, versus 33.3 %) and per woman after a mean number of ≈ 1.5 fresh D-IVF cycles (38.0 % versus 50.6 %) were also lower in the lesbian group although differences were not significant. These results contrast with the similar success percentages obtained in frozen/thawed D-IVF cycles. In particular, the pregnancy percentage per cycle was 31.6 % versus 31.7 % and the pregnancy percentage per woman after a mean number of ≈ 2 cycles was 63.3 % versus 64.7 % in the lesbian and heterosexual group, respectively. Likewise, live-birth percentage per frozen/thawed D-IVF cycle (24.5 % versus 23.1 %) and per woman after a mean number of ≈ 2 frozen/thawed D-IVF cycles (49.0 % versus 47.1 %) were also similar in pair-bonded lesbians and pair-bonded heterosexuals.

Sampling bias, small sample sizes and inadequate use of statistical methods to control for the effects of influential covariates may explain discrepancies in success percentages of pair-bonded lesbians and pair-bonded heterosexual women reported by Nordqvist *et al.* [29] after D-IUI, fresh D-IVF and frozen/thawed D-IVF.

### Success percentages of gays in gestational-surrogacy programs

Like in lesbians after D-IUI, gays entering into gestational-surrogacy programs tend to have higher success percentages than heterosexual men (Table 2). Grover *et al.* [21] reported that 56.8 % (21/37) of surrogate women recruited by pair-bonded gays and 50.0 % (4/8) of surrogate women recruited by single men (7 homosexuals and one heterosexual) succeeded in achieving a pregnancy after a maximum of either two fresh cycles or one fresh and one frozen transfer cycle. No significant differences in delivery percentages per surrogate woman between the group of pair-bonded gays/single men and a control group of heterosexual couples were evidenced (55.6 % versus 48.9 %). Of note, this study did not control for the potential negative effects of male reproductive aging on semen parameters and embryo/fetal development (for review, see [39]). Furthermore, the study did not provide any information about epidemiological data of heterosexual couples, oocyte donors and gestational carriers including ovarian stimulation protocol and medical indications for using gestational surrogacy.

These deficiencies were partially amended in a recent study by the same group [22]. This study reported the largest gestational surrogacy series published so far (333 consecutive gestational surrogacy cycles including cycles from most of the pair-bonded gays and single men previously reported by Grover *et al.* [21]). On this occasion, the medical indications of heterosexual couples for using gestational surrogacy were specified. Notwithstanding, data analysis were either not controlled for ovarian stimulation protocol and age of intended parents, oocyte donors and gestational carriers. In addition, the group of heterosexual couples was not homogeneous. In fact, intended heterosexual parents used donor oocytes in 39.5 % (107/271) of cycles (mean age of oocyte donors: 26.2 years) whereas in the remaining 60.5 % (164/271) of cycles (mean women’s age: 36.1 years) they used their own (autologous) oocytes. Furthermore, the group of heterosexual couples included a total of 17 cycles in which embryos were concurrently transferred to both the intended-parent woman and the gestational carrier. In contrast, all the 52 pair-bonded gays/single men entered into the study used exclusively donor oocytes and gestational carriers.

The sampling asymmetries/biases between groups may explain, at least in part, the higher, although not statistically significant, pregnancy percentage per cycle evidenced in the “pair-bonded gays/single men” group (59.7 %) compared with a “failed to carry” (50.0 %) and a “cannot carry pregnancy” (54.0 %) group. Likewise, the “pair-bonded gays/single men” group displayed lower miscarriage percentages per pregnant surrogate woman (10.8 %) than the “failed to carry” (25.8 %) and the “cannot carry pregnancy” (20.0 %) group, although differences among groups were not statistically significant.

### Concluding remarks

The present review shows that lesbians after D-IUI and gays entering into gestational-surrogacy programs tend to have higher success percentages than heterosexuals. This trend is not surprising if we take into account that lesbians usually enter into D-IUI programs and gays into gestational-surrogacy programs to fulfill their wishes to have and raise their own offspring and create families, not because they are infertile. However, the only single study published so far on success percentages of lesbians after D-IVF [29] provides puzzling results. In particular, data show that pair-bonded lesbians have lower pregnancy and live-birth percentages than pair-bonded heterosexual women in fresh D-IVF cycles. In contrast, pregnancy and live-birth percentages are similar in frozen/thawed D-IVF cycles. Of note, all the reports reviewed in the present study are methodologically flawed because of sampling bias, small sample sizes and inadequate use of statistical methods to control for the effects of influential covariates including age, smoking habits, previous gynecological problems, hormonal stimulation type and protocol, and number of prior treatment types and pregnancies/deliveries. Clinicians, reproductive biologists and editors of fertility/infertility journals should make efforts to prevent these deficiencies in future data reporting.

## Abbreviations

CC: Clomiphene citrate
D-IUI: Intrauterine insemination with donor spermatozoa
D-IVF: In-vitro fertilization with donor spermatozoa
FSH: Follicle stimulating hormone
HIV: Human immune virus
hMG: Human menopausal gonadotropins
ICSI: Intracytoplasmic sperm injection
IUI: Intrauterine insemination
IVF: In-vitro fertilization
PCOS: Polycystic ovary syndrome
